# Supplementary material for: Study of Liquid-Based Cytology Using Next-Generation Sequencing as a Liquid Biopsy Application in Patients with Advanced Oncological Disease
Source: Biomedicines. 2023 May 29;11(6):1578. doi: 10.3390/biomedicines11061578 (PMC10295144; doi:10.3390/biomedicines11061578)
Supplement: Supplementary file 1 [file biomedicines-11-01578-s001.zip › biomedicines-2337675-supplementary.pdf]

Supplementary Table S1. Genes interrogated by Oncomine™ Precision Assay panel

45 genes for the detection of hotspot mutations						
AKT1	*AKT2*	*AKT3*	*ALK*	*AR*	*ARAF*	*BRAF*
CDK4	*CDKN2A*	*CHEK2*	*CTNNB1*	*EGFR*	*ERBB3*	*ERBB2*
ERBB4	*ESR1*	*FGFR1*	*FGFR2*	*FGFR3*	*FGFR4*	*FLT3*
GNA11	*GNAQ*	*GNAS*	*HRAS*	*IDH1*	*IDH2*	*KRAS*
MAP2K1	*MAP2K2*	*MET*	*MTOR*	*NRAS*	*NTRK1*	*NTRK2*
NTRK3	*PDGFRA*	*PIK3CA*	*PTEN*	*RAF1*	*RET*	*ROS1*
SMO	*TP53*					

14 genes for the detection of copy number alterations						
ALK	*AR*	*CD274*	*CDKN2A*	*EGFR*	*ERBB2*	*ERBB3*
FGFR1	*FGFR2*	*FGFR3*	*KRAS*	*MET*	*PIK3CA*	*PTEN*

19 genes for the detection of gene fusions involving intergenic and intragenic regions						
ALK	*AR*	*BRAF*	*EGFR*	*ESR1*	*FGFR1*	*FGFR2*
MET	*NGRG1*	*NTRK1*	*NTRK2*	*NTRK3*	*NUTM1*	*RET*
ROS1	*RSPO2*	*RSPO3*				
